# Current Status and Potential Applications of Underexplored Prokaryotes

**DOI:** 10.3390/microorganisms7100468

**Published:** 2019-10-18

**Authors:** Kian Mau Goh, Saleha Shahar, Kok-Gan Chan, Chun Shiong Chong, Syazwani Itri Amran, Mohd Helmi Sani, Iffah Izzati Zakaria, Ummirul Mukminin Kahar

**Affiliations:** 1 Faculty of Science, Universiti Teknologi Malaysia, Skudai 81310, Johor, Malaysia; salehas@utm.my (S.S.); cschong@utm.my (C.S.C.); syazwaniitri@utm.my (S.I.A.); helmisani@utm.my (M.H.S.); 2 Division of Genetics and Molecular Biology, Institute of Biological Science, Faculty of Science, University of Malaya, Kuala Lumpur 50603, Malaysia; kokgan@um.edu.my; 3 International Genome Centre, Jiangsu University, ZhenJiang 212013, China; 4 Malaysia Genome Institute, National Institutes of Biotechnology Malaysia, Jalan Bangi, Kajang 43000, Selangor, Malaysia; iffahizzati@nibm.my

**Keywords:** ex situ diffusion bioreactor, ichip, metagenome-assembled genome, rare microorganisms, shotgun metagenome sequencing, unculturable bacteria

## Abstract

Thousands of prokaryotic genera have been published, but methodological bias in the study of prokaryotes is noted. Prokaryotes that are relatively easy to isolate have been well-studied from multiple aspects. Massive quantities of experimental findings and knowledge generated from the well-known prokaryotic strains are inundating scientific publications. However, researchers may neglect or pay little attention to the uncommon prokaryotes and hard-to-cultivate microorganisms. In this review, we provide a systematic update on the discovery of underexplored culturable and unculturable prokaryotes and discuss the insights accumulated from various research efforts. Examining these neglected prokaryotes may elucidate their novelties and functions and pave the way for their industrial applications. In addition, we hope that this review will prompt the scientific community to reconsider these untapped pragmatic resources.

## 1. Introduction

Ample numbers of prokaryote (bacteria and archaea) phyla have been described. The modern classification of prokaryotes involves polyphasic characterizations [1]. The state-of-the-art methods—for instance, phylogenomics, average nucleotide identity, and percentage of conserved proteins—are becoming popular for assisting the delineation of prokaryote genera [2,3,4]. To date (September 2019), more than 3800 prokaryotic genera have been published directly in the International Journal of Systematic and Evolutionary Microbiology (IJSEM) or were included in the validation list, under the Rules of the International Code of Nomenclature of Bacteria [5,6]. The LPSN database (List of Prokaryotic names with Standing in Nomenclature database; http://www.bacterio.net) archives all validly published names of prokaryotes. The BacDive bacterial metadatabase has collected data on more than 80,500 strains, including 13,500 type strains from 34 bacterial and three archaeal phyla [7]. The World Data Centre for Microorganisms (WFCC-MIRCEN; http://www.wdcm.org) is another data center for microbial resources. The WFCC Global Catalogue of Microorganisms (GCM) has catalogued 447,444 strains from 48 countries and regions. 

**Figure 1:**
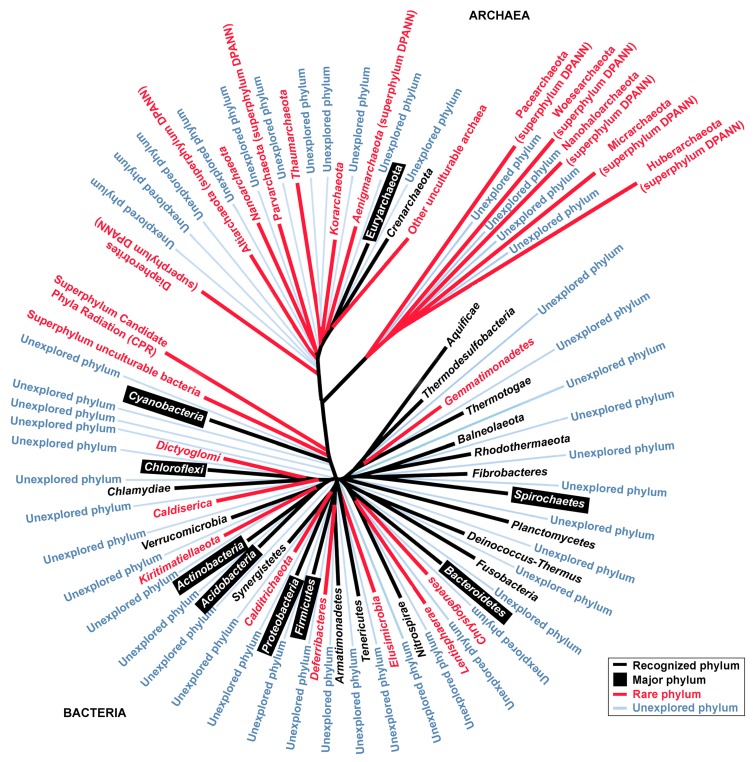
Illustration of 16S rRNA phylogenetic tree (phyla). The representatives were aligned using ClustalW, and a maximum-likelihood tree with a bootstrap value of 1000 replicates was built within the MEGA6 package [27]. The transformed radiation tree was redrawn using the FigTree version 1.4.4 program. The number of 16S rRNA sequences for each phylum is provided in Table 1.

The NCBI taxonomy and Silva databases possess the highest numbers of deposited 16S rRNA sequences for the bacterial phyla Proteobacteria, Firmicutes, Actinobacteria, and Bacteroidetes (Table 1). Additionally, these phyla have abundant culture representatives (Figure 1) [7,8]. The primary genera listed in Table 2 have been mentioned in >868,000 related articles in the Scopus database. In addition, the top ten strains with the most relevant patents are listed in Table 3. 

Currently, there are three main strategies for genome sequencing. The first approach is whole-genome sequencing (WGS) of cultured prokaryotes using Illumina, PacBio, Nanopore, Qiagen, BGISEQ, IonTorrent, or other sequencers. Some of these platforms are more frequently used than others. There are at least 180,312 registered WGS sequencing projects and 250,398 analysis projects in the Genomes OnLine Database (GOLD) of the Joint Genome Institute (JGI) (September 2019) [9]. Proteobacteria (51%), Firmicutes (29.8%), and Actinobacteria (12.1%) account for 92.9% of the sequenced bacterial phyla. For archaeal phyla, intensive sequencing has been performed for Euryarchaeota (59.5%), Crenarchaeota (24.1%), and Thaumarchaeota (13.5%). However, in comparison to that given to the major phyla mentioned above, relatively little attention has been given to other phyla (Table 1, Figure 1). Due to the increasing amount of genomic data, and in order to maintain a certain quality of genome description, the Genomic Standards Consortium (GSC; http://gensc.org) has proposed minimum standards, namely the Minimum Information about a Genome Sequence (MIGS) [10]. Readers can refer to the latest standard guidelines on the official webpage of EMBL-EBI (https://www.ebi.ac.uk/ena/submit/mixs-checklists). Readers are referred to review articles that summarize achievements in the genome sequencing of culturable bacteria [11,12,13], and to specific reviews on thermophiles [14,15] or bioinformatics tools for microbial genomes [16].

The second primary strategy to genome sequencing is shotgun metagenome sequencing, which can generate DNA reads directly from an environment without the need to culture individual colonies. The whole process involves environmental DNA (eDNA) extraction, amplification, and sequencing by a high-throughput sequencing system. The generated DNA reads can be imagined as mixed-up pieces from different boxes of jigsaw puzzles. In this analogy, each box represents one bacterium or archaeon. DNA fragments generated by the sequencer are grouped (binned) accordingly and assembled into contigs using bioinformatics simulations. The qualified and approved bins are known as metagenome-assembled genomes (MAGs) [17,18]. At the time of writing this manuscript (September 2019), the JGI GOLD has recorded 11,723 MAG projects. The general standard or guidelines for this approach are available in the Minimum Information about a Metagenome-Assembled Genome (MIMAG) [19]. 

**Figure 2:**
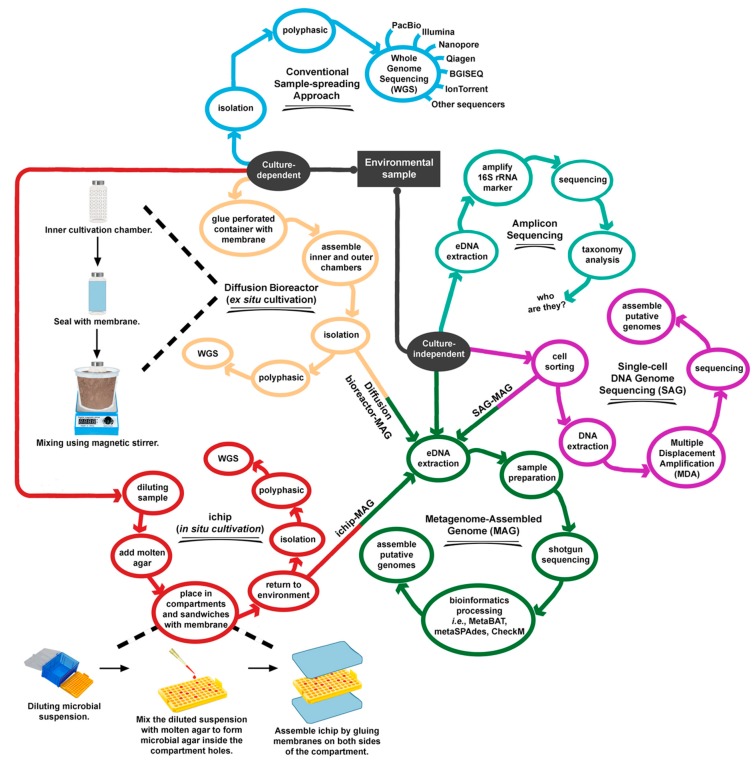
Overall processes of discovery of cultured and unculturable prokaryotes.

The third main strategy to genome sequencing is single-cell DNA genome sequencing (SAG), which is another culture-independent approach. In comparison to WGS and MAG data, a lower amount of SAG data is deposited in the JGI GOLD (2168 projects). SAG involves disengaging single cells using a microfluidic system or similar, extracting DNA, performing DNA amplification using Multiple Displacement Amplification technology, constructing sequencing libraries, DNA sequencing, and assembling the reads into contigs. Examples of SAG-related articles are provided in [20,21]. Researchers are required to comply with most, if not all requirements of Minimum Information about a Single Amplified Genome (MISAG) before submitting SAG sequences to databases [19]. The JGI earlier funded a project to develop a microfluidic-based mini-metagenomic method [22], an approach that integrates SAG and MAG (Figure 2). Using the new method, the research team successfully extracted and assembled new genomes from hot spring water samples [18,22,23]. Nevertheless, SAG applications have been mostly focused on clinical specimens in oncology, immunology, neurobiology, and prenatal diagnosis. Therefore, SAG is not covered in detail in this article but has been explained in earlier publications [18,24,25,26].

In this review, we focus our discussion on the current status and potential applications of underexplored prokaryotes. Herein, we define underexplored prokaryotes as (i) unculturable prokaryotes, and (ii) genera consisting of limited species listed in the LPSN database.

## 2. Underexplored Prokaryotes 

### 2.1. Definition of Underexplored Prokaryotes

The word ‘rare’ is loosely defined in microbiological, taxonomical, and ecological perspectives. Microbiologists often refer to rare or underexplored prokaryotes as (i) culturable genera with limited type strains, (ii) unculturable microorganisms under laboratory conditions, or (iii) prokaryotes that present a minority population in the environment. The majority of the prokaryotes present in a sample are mostly uncultivable [13,30,31,32]. These underexplored prokaryotes are often referred to as ‘microbial dark matter’, or ‘unculturable bacteria’. Rare prokaryotes are also microorganisms present as minorities in the environment whose abundance can be as low as <0.001% of the total population [33]. 

### 2.2. Reasons for Analysing Underexplored Prokaryotes 

Below, we give three main motivations behind the interest of research groups working in the field of underexplored prokaryotes. The first motivation is obtaining fundamental knowledge and completing a bigger picture. Trees constructed using currently known cultured taxa are only the ‘tip of the iceberg’ of the entire group of existing prokaryotes (Figure 1). Researchers accept that many prokaryotes are still hidden in the tree of life. Cultivation-independent methods (i.e., 16S rRNA amplicon sequencing, MAG, and SAG) can further expand our understanding of domain bacteria beyond those that have been cultivated [34]. Studying rare prokaryotes generates new scientific information that unveils their uniqueness. Some of these underexplored prokaryotes have atypical characteristics; for instance, unique cell wall structures [35]. A recent report suggests that rare prokaryotes play a crucial buffering role in biotic community membership and stability, besides acting as a genetic reservoir in the face of environmental perturbation [36]. It is well understood that the marine microbial community plays an enormous role in recycling global nutrients [37], and most currently established biological pathways are built on well-known prokaryotes. However, it is less understood that certain underexplored prokaryotes also have biological roles in the environment, in particular in geochemical cycles of carbon, nitrogen, and sulfur [34,38,39]. In the past, researchers only examined the effects of abiotic factors on overall microbial diversity, particularly the predominant taxa [40,41,42,43]. Researchers have started to investigate how environmental factors affect the diversity of the rare prokaryotes instead of the major microorganisms [44]. As we broaden horizons of underexplored prokaryotes, genomic evolution becomes better understood, and missing links in gene transfer are discovered, so that revising the tree of life may be possible.

The second primary motivation of working in the field of underexplored bacteria is their association with health and diseases. Researchers suspect that certain underexplored bacteria are related to infections in humans, animals, or plants. For example, the candidate (or *candidatus* in Latin) *Liberibacter asiaticus* is pathogenic to potato, carrot, tomato, and citrus [45]. The threat from these bacteria is so severe that the US Environmental Protection Agency (EPA) is currently in the process of allowing citrus growers to spray antibiotics to control pathogenic *Liberibacter* species [46]. In humans, the growth of an underexplored prokaryote, namely candidate *Borkfalki ceftriaxensis*, was observed following antibiotic treatment with ceftriaxone.

Additionally, it is now understood that other than viruses, bacteriophages, fungi, and bacteria, the candidate phyla radiation (CPR) superphylum group of bacteria interact with each other in the human oral environment and collectively may impact human health [34]. On the other hand, the search for new antibacterials also needs to continue. It needs to be determined whether underexplored microorganisms are important sources of new antibacterial agents. The US Government Defense Advanced Research Projects Agency (DARPA) conceptualized the Pathogen Predators Research Program with an enacted budget of 3.4 billion USD for 2019. One of DARPA’s projects was searching for predatory bacteria against pathogens [47]. To date, *Bdellovibrio bacteriovorus* (from a genus consisting of four type strains) and *Micavibrio aeruginovorus* (from a monotypic genus) are the only predatory bacteria known to prey upon >100 different human pathogens [48,49,50]. 

The third primary motivation is the new applications—Blue Ocean Strategy. Protein sequences of underexplored prokaryotes exhibit low similarities to other well-established proteins. The exploration of underexplored prokaryotes and their macromolecules and natural products is regarded as the ‘blue ocean strategy’ in science (a term coined from a book of the same title by Mauborgne and Kim [51]). The author defined the ‘blue ocean strategy’ as “the simultaneous pursuit of differentiation to open up a new market space and create new demand”. Therefore, discovering new strains or native and novel macromolecules may establish new, if not, better applications. In some reports, the total number of genes annotated as hypothetical proteins represent half of the total number of genes [52]. Some archaeal genomes had up to 80% hypothetical proteins [53]. It would be interesting to identify new potential applications of these unknown genes. In addition, exploring rare prokaryotes is an opportunity for an offensive patent strategy of potentially lucrative novel bioeconomy. 

### 2.3. Why Are Great Proportions of Prokaryotes Unculturable?

Researchers blame the prokaryotes since much about them remains ‘black box information’ or non-reproducible under the designated laboratory setup. For example, (i) cells may need unknown special culture requirements: uncommon nutrients, a narrow temperature or pH range, or unusual compounds to support their growth. (ii) Cells from the environment are in the dormancy stage, and none of the resuscitation approaches can make them appear on the Petri dish, and (iii) having a smaller genome size causes most candidate CPR to lack numerous biosynthesis pathways, to be absent of ATP synthase, and to lack the electron transport chain complex [54]. As a result, Dombrowski et al. [55] concluded that CPR and DPANN members are unable to grow individually but, rather, rely on resources contributed by neighboring bacteria through symbiosis, parasitism, or other relationships. Despite such biological interactions also existing in culturable microorganisms [56], CPR and DPANN members are relatively more delicate. 

### 2.4. How Should Underexplored Prokaryotes Be Cultured? 

It is not impossible to isolate underexplored prokaryotes from the environment. From the authors’ analysis using data from the Deutsche Sammlung von Mikroorganismen und Zellkulturen (DSMZ), at least 1000 genera are single-species representatives (monotypic or monospecies) which were once categorized as ‘unculturable’ (Table 4). Although some monotypic strains require stringent isolation or cultivation strategies, many of the bacteria are not at all demanding to cultivate. Although the suggestions provided hereafter are not new, however, microbiologists in their early career may be unaware of them. 

The first suggestion is paying attention to isolation and growth preparation. Autoclaving agar with phosphate remarkably lowers the total number of colonies that grow on the agar plates [57]. Agar contains some inhibitors that prevent the growth of some prokaryotes; gelrite is a better solidification agent. The enrichment of any environmental sample (e.g., soil or sediment) using atypical chemicals may enhance the probability of isolating rare prokaryotes. For example, Aanderud [58] used heavy water (H_2_^18^O) to rewet soil samples. Interestingly, after introduction of heavy water, the abundance of initially recognized rare microorganisms increased considerably, from hardly detectable to the dominant proportion of the community.

The second suggestion is: reconsider sampling sites. In order to increase the success rate of discovering undomesticated species, individuals who conduct the sampling should perhaps consider using some hard-to-reach locations where human activities are at a minimum. For example, hydrothermal vents in ocean basins, boiling geothermal springs, and caves, such as those at Mount Roraima in South America; rainforests, such as the deep Amazon or Malaysia’s Kinabalu National Park; parched dry places, such as the Atacama Desert colourful lakes, such as those of Indonesia’s Kelimutu volcano; or deep-cold places, such as Antarctica (below 2.75 km in depth). Members of the Extreme Microbiome Project are interested in poly-extremophiles, for example, thermophiles in the Hell gas crater of Turkmenistan, and halophiles in Lake Hiller of Australia [59]. Readers are referred to a recent review article that discusses current knowledge on extremophiles [60]. 

The third suggestion is modifying cultivation strategy. (i) In situ cultivation: If possible, researchers could consider using a device such as isolation chips (ichips) to maximize the number of individual colonies [61,62]. The idea behind the ichip is to dilute the environmental samples using molten agar, trapping each cell into a hollow compartment sealed with a membrane, and returning the ichip to the environment (Figure 2). Using the ichip, a new class of antibiotics (teixobactin) was discovered from an unculturable bacterium, *Eleftheria terrae* [47]. (ii) Ex situ cultivation: Recently, Chaudhary et al. [63] described a new bacterial cultivation device termed as a diffusion bioreactor (Figure 2). Using this approach, the authors isolated 35 previously uncultured strains from phyla Proteobacteria, Firmicutes, Actinobacteria, and Bacteroidetes. The diffusion bioreactor is more flexible than that of the in situ ichip method. As the diffusion bioreactor is performed in the laboratory, researchers have a better selection of abiotic or experimental parameters, for instance, pH, temperature, moisture, nutrients, and timing. Lately, Sun et al. [64] discussed several strategies to improve archaeal cultivation.

### 2.5. Exploring Unculturable Prokaryotes Using Metagenome-Assembled Genomes (MAG)

Even with intensive isolation efforts, a certain proportion of prokaryotes escape cultivation—for example, candidate phyla, unculturable superphylum, such as the bacterial CPR and archaeal DPANN, and other unexplored or unknown phyla (Figure 1). The exact number of unexplored phyla is unknown. Examples of a few candidate phyla are listed in Table 1, and this number is expected to rise. The naming of uncultured taxa could be confusing due to inconsistencies in nomenclature [92], dynamic updates and renaming, and differences of information in major databases, for instance, those of the NCBI Taxonomy, Silva, and Genome Taxonomy Database (Table 1) [2,8]. 

To date, unculturable prokaryotes bacterial CPR membership has been conferred to more than 70 Candidate phyla [92]. There are many putative archaea phyla identified via culture-independent approaches [53,64]. The cumulative 16S rRNA amplicon sequencing and genomic data have led to the proposal of several archaea superphyla. For instance, DPANN is the largest archaea superphyla with >24,000 deposited 16S rRNA sequences listed in NCBI taxonomy and Silva databases (September 2019). The readers can refer to a recently published review article on DPANN (candidate Aenigmarchaeota, Altiarchaeota, Diapherotrites, Hadesarchaeaeota, Huberarchaeota, Micrarchaeota, Nanohaloarchaeota, Pacearchaeota, Parvarchaeota, and Woesearchaeota) for more insights on the genomic features, lifestyle, and evolution [55]. Other unculturable archaea superphyla include Asgard (also known as Asgardaeota) and TACK (also known as Proteoarchaeota) [64]. The TACK superphyla group consisted of Nitrosopumilales, Nitrosotalea, Nitrosophaerales, Nitrospcaldales, Geothermarchaeota, Aigarchaeota, Bathyarchaeota, Marsarchaeota, Nezhaarchaeota, Geoarchaeota, and Verstraetearchaeota. However, the TACK is undergoing dynamic membership updates and renaming, and the taxonomy affiliation of certain members are uncertain. 

MAG is one of the most effective ways to glimpse the genomes of unculturable prokaryotes. Anantharaman et al. [33] described 47 newly discovered phyla using 2540 reconstructed MAGs and elucidated that microbiome biological pathways are cross-linked. Danczak et al. [93] recently discussed the putative functions of 32 already known CPR Candidate phyla in carbon processing and nitrogen cycling. An impressive large-scale reconstruction of 7903 MAGs was performed, which provided the first genomic representatives of new rare candidate bacteria and archaea [17]. Accordingly, all the assembled genomes have at least 50% completeness, and nearly half of the total MAGs are ≥90% complete with less than 5% contamination. In a separate study, MAG analyses of two archaea from a hyperthermal hot spring identified unrecognized methane-metabolising sequences outside of the phylum Euryarchaeota [94]. In another work, Kadnikov et al. [95] recovered a complete genome of the candidate phylum BRC1 using MAG analysis of a deep subsurface thermal aquifer. For more information, readers can refer to a recent review article by Quince et al. [96] that covers experimental design, sampling, and analysis using shotgun metagenomics for MAG. A summary of MAG-related research and bioinformatics tools is given in Table 5. CheckM is a tool used to determine genome completeness and identify contaminant sequences [97].

## 3. Potential Applications of Underexplored Prokaryotes

### 3.1. Potential Applications of Culturable Rare Prokaryotes 

A recent study has shown that depths of 6−11 km below sea level are heavily polluted by microplastic (fragment size ≤ 5 mm) [113]. A recent review article summarized current milestones in the use of microbial enzymes for modifying or degrading different categories of plastics, for instance, polyurethane and polyethylene terephthalate (PET) [114]. Unfortunately, the degradation process of highly crystallized and packed plastics is still considered slow and ineffective. Readers are advised to read a review on marine microbial adaptation, interaction, and degradation of microplastics [115]. The biodegradation of recalcitrant plastics involves various groups of enzymes; including cutinases, laccases, manganese peroxidases, lignin peroxidases, alkane hydroxylases, tannases, hydroquinone peroxidases, ureases, esterases, lipases, proteases, and polyester hydrolases, although each with a different extent of degradations [114]. Enzymes from various genera have been described to have some form of plastic degrading ability. *Delftia*, *Thermobifida*, and *Ideonella* being among the genera with limited type strains [114]. *Ideonella sakaiensis* is able to use PET as carbon source [116]. Enzymes from *I. sakaiensis* and *Thermobifida fusca* have been extensively examined with regards to the aspects of enzyme catalytic mechanism, structure, and function [117,118,119,120]. In nature, PETases (PET hydrolase) are esterase active on ester bonds [116,120]. In a separate work, Danso et al. (2018) mined 853 gene sequences using a metagenomics approach and showed that PETases are mainly distributed in culturable Actinobacteria, Proteobacteria, and Bacteroidetes [121], and among the sources, *Caldimonas* and *Methylibrium* can be further explored. Additionally, PETase homologues are also found in other groups of bacteria [118].

Efforts have been invested in harnessing rare marine Actinobacteria. Examples of underexplored *Actinobacteria* are *Actinoalloteichus*, *Salinispora*, *Marinactinospora*, and *Actinosynnema*. These prokaryotes produce active compounds with cytotoxic, antibacterial, antifungal, and antimalarial activity [122]. The phylum Actinobacteria is one of the largest phyla in terms of total isolates and sequences deposited in databases (Figure 1, Table 1). However, the isolation and discovery of new Actinobacteria species are continuing. In a large-scale screening, Idris et al. [123] elucidated that 16% of the total sequence reads associated to this phylum could only be assigned up to class level based on the EzTaxon-e database, and these new taxa had relatively low similarity to already-described Actinobacteria genera. 

Lambrechts and Tahon [124] recently summarized the progress of Antarctic microbiology studies and listed many monotypic genera of rare bacteria. More attention should be given to psychrophilic microorganisms, as their enzymes are of potential use in biotechnology, particularly for applications that require lower temperatures [125]. There are many underexplored psychrophilic prokaryotes; for example, a monotypic *Raineyella antarctica* was recently isolated and sequenced [126]. Sheridan et al. [127] described *Rhodoglobus* 16 years ago while Li et al. [128] first reported monotypic *Marisediminicola antarctica* ca. 10 years ago. Unfortunately, neither genera have received much attention. Few researchers are interested in studying psychrophiles, possibly because many of these bacteria grow slowly and most of their proteins (enzymes) are sensitive to higher temperatures.

Industrial enzymes, such as hydrolases for starch, cellulose, hemicellulose, lipids, and esters, are biocatalysts that have been extensively targeted using culture-based approaches. Many rare bacteria type strains harbour interesting genes, and a few are listed here. *Melioribacter roseus* is a facultative anaerobe and a thermophile that is rich in various glycosyl hydrolase (GH) genes [129,130,131]. The bacteria *Siansivirga zeaxanthinifaciens*, a mesophile, and *Alkalitalea saponilacus*, an anaerobe and alkalophile, also harbour a broad range of GHs [132,133,134,135]. All the bacteria mentioned above are monotypic of their respective genus. *Jeotgalibacillus* is another example of an underexplored genus whose enzymes may be of use [136]. Liew et al. [137] recently elucidated that *J. malaysiensis* produces glucose-tolerant β-glucosidase. 

*Rhodothermaceae* (thermophilic), *Rubricoccaceae* (mesophilic), *Salisaetaceae* (halophilic), and *Salinibacteraceae* (halophilic) are families of the order Rhodothermales. To date, this order consists of ten genera, each with no more than three species. The order is small, having only 16 validly described type strains. *Rhodothermus* spp. produce important cellulosic and hemicellulosic hydrolases [138,139]. However, none of the other genera in the order Rhodothermales have been studied for counterpart enzymes. Lately, Park et al. [140] isolated *Roseithermus sacchariphilus* gen. nov., sp. nov. (strain MEBiC09517^T^) from sediment collected from a coastal area. Due to the high genome-to-genome similarity, our isolated bacterium *Rhodothermaceae* RA is the subspecies of strain MEBiC09517^T^ [141,142]. The bacterium *R. sacchariphilus* RA has 57 glycosyl hydrolase sequences. We have cloned and characterized two novel xylanase enzymes [143,144]. For one of the novel enzymes that we characterized, the protein sequence showed less than 50% sequence identity to well-characterized counterpart enzymes. The authors believe that all members of the order Rhodothermales should be given more consideration for the discovery of enzymes.

The International Society of Rare Sugar (http://www.isrs.kagawa-u.ac.jp) has defined rare sugars as monosaccharides and their derivatives that rarely exist in nature [145]. These fine chemicals (i.e., D-allose, D-psicose, D-gulose, D-sorbose, and L-ribose) are valuable and have numerous applications in food products and sweeteners, pharmaceuticals, and agriculture [145,146,147]. The conversion of natural sugars to rare sugars involves several enzymatic reactions involving isomerases, epimerases, and oxidoreductases [145]. The majority of enzymes already applied in the industry have been sourced from cultured bacteria. To the best of our knowledge, attempts of finding underexplored culturable prokaryotes as well as gene-mining using a metagenomic approach to produce rare sugar are scarce [147].

Many monotypic bacteria remain to be discovered and could provide insights into fundamental sciences and offer possible biotechnological applications. Table 4 summarizes some monotypic bacteria with genome information. Other records of monotypic bacteria and genera are available on the LPSN database and Wikipedia (http://www.wikipedia.org; keyword: monotypic bacteria. Note: the list may not be auto updated). Readers who wish to have access to a periodically updated list of prokaryotes can refer to *Prokaryotic Nomenclature* on the official webpage of DSMZ. 

### 3.2. Potential Research Topics in Unculturable Prokaryotes

For culturable rare prokaryotes, we store the cells on Petri dishes or freeze them as stock cultures. This strategy is different from exploiting resources from unculturable prokaryotes since we do not have cells of unculturable prokaryotes. Researchers only retain extensive digital information (contigs or scaffolds, annotated genes, or sequences of proteins) and a limited volume of eDNA. The easiest way to explore biological resources (genes and proteins) mined in MAGs or SAGs is by synthesizing the complete genes using commercial service providers, cloning the genes in a suitable vector, expressing the genes as recombinant proteins, examine the biochemical functions, or analyze the protein structures by methods such as X-ray crystallization. Although synthesizing several genes is affordable for most laboratories, this workflow is not flawless as the selection of the genes is pivotal. It is not always possible to pinpoint the right targeted genes due to knowledge limitations, or the expressed recombinant proteins are neither active nor function as anticipated.

Ocean and coastal bodies are the most significant reservoirs of diverse microbes and important sources for the discovery of prokaryotes. Tara Oceans is a well-known global ocean expedition involving hundreds of researchers. MAG information from this project is publicly available [107]. Other MAG data obtained from various environments are also deposited in public databases, such as the NCBI. By exploring putative genes of interest generated in-house or from sources such as the Tara Oceans expedition, researchers will have ample genes that encode biocatalysts or macromolecules that may be applicable in biotechnology. Examples of metagenome-derived biocatalysts and proteins, both from the common and underexplored prokaryotes, are listed hereafter; haloalkane dehalogenase, esterase, β-glucanase, keratinase, exonuclease, and endoglucanase [148,149,150,151,152,153]. Other examples of recent work using MAGs to mine biocatalysts and proteins are listed in [150,154]. Readers are encouraged to read some excellent review articles on the topic of MAGs [155,156].

## 4. Limitations and Future Directions of Prokaryote Discovery 

The authors agree with the opinion of Garza and Dutilh [157] that scientists have only scratched the surface of the vast microbial world. The total number of phyla in the prokaryotic domain remains unknown (Figure 1). This review does not intend to exaggerate the importance of underexplored prokaryotes. We acknowledge that prokaryotes such as *Bacillus* are still one of the best workhorses for industrial applications. One needs to understand that there are many obstacles and limitations to exploring rare prokaryotes, despite the rewards it may offer, and a few will be discussed here. 

Using single-cell genome amplification to explore unculturable prokaryotes is not straightforward. Hedlund et al. [158] reported that SAG is not an ideal approach if the environmental sample is too complicated. However, there have been a few successful applications. In most cases, genome assembles derived from SAG is shorter than MAG, depending on the type of samples [18,21,159]. On the other hand, SAG yields more focused reads than the wider MAG. As such, methods integrating SAG and MAG, such as the JGI’s innovative microfluidic-based mini-metagenomic method, offers a more accurate complete genome [22]. However, not all laboratories have the resources to use microfluidic technology. 

The invention of the ichip is interesting and nothing short of ingenious. Furthermore, all laboratories can convert affordable plastic pipette tip racks into a homemade ichip. According to the descriptions in Berdy et al. [160], it is possible to obtain a single type of cell or combination of a few strains in each chamber. We speculate that pairing the ichip with MAG would be much easier and cheaper than SAG–MAG methods (Figure 2). However, this concept has not been experimentally proven. 

Prokaryote genomes vary in size; for example, candidatus Carsonella ruddii has a tiny size of 0.17 Mb, while the *Sorangium cellulosum* strain So0157-2 has a very large size of 14.78 Mb [161,162]. The mean genome size for prokaryotes is ~3.7 Mb (https://www.ezbiocloud.net) [163]. Based on statistical analysis, many Actinomycetes strains that produce important secondary metabolites have large genomes, approximately >8 Mb [122]. According to the article, the coding capacity increases with genome size [122]. It is not uncommon that prokaryotes with genomes smaller than 8 Mb also exhibit a complete set of secondary metabolite gene clusters as well as other important industrially-applicable proteins. However, unculturable CPR and the DPANN superphyla have very small genomes because they have minimal biosynthetic capacities [52,54,55]. Therefore, some unculturable prokaryotes, especially those with extraordinarily small genomes, may encode lesser industrially applicable proteins, thus, limiting their exploitation in biotechnology. The genus *Bacillus* is a favorite source of industrial enzymes because it has a sufficiently large genome and, thus, produces a broad range of proteins, compared to its counterpart *Anoxybacillus*, whose genome is approximately 50% shorter. Some of the commonly found genes, for instance enzyme CGTase, is present in *Bacillus* and *Geobacillus*, but absent in taxonomically-related *Anoxybacillus* due to genome shrinking [164,165]. Nevertheless, this does not mean that prokaryotes with a small genome have no potential use. For example, *Caldicellulosiruptor saccharolyticus* is an excellent thermophilic candidate for H_2_ production and plant biomass degradation, despite its genome (2.9 Mb) being smaller than the mean size of prokaryote genomes [166]. Many hyperthermophilic archaea in general have smaller genomes, for instance, the genome size for *Pyrococcus* spp. is ~1.9 Mb [167]. DNA polymerase from *Pyrococcus* is one of the high-fidelity enzymes that has been long commercialized by New England Biolabs. 

We want to emphasize that MAG is not a magic staff. MAG is still not a common approach that all laboratories can perform. Novel sequences can be found from MAGs, even when a pure culture is not available. It is possible that the assembly of reads is imperfect, and this can create an artefact gene. The translated recombinant protein can malfunction due to a mutation at important positions due to the artefact in the gene. Unfortunately, even with properly assembled genes, for instance, those identified in high-quality WGS, various sophisticated persistent problems remain unsolved; for example, failure due to protein misfolding in the expression hosts. In addition, determining the roles of hypothetical proteins (assembled in WGS, MAG, or SAG) is relatively more costly and time-consuming. Deducing the protein’s function may require additional work, such as transcriptomic and in silico functional prediction. The crystallization of hypothetical proteins to identify clues from the protein structure is another potential method. Readers can refer to recent work on the discovery of hypothetical proteins [168,169,170,171]. The numbers of hypothetical proteins will expand rapidly as more underexplored genomes are sequenced; however, the speed to understand proteins’ functions remain sluggish [53]. 

## 5. Conclusions

We believe that all creatures, including underexplored prokaryotes, have the potential for as-yet-unknown purposes. The cultivation of underexplored prokaryotes is somewhat challenging; yet, with the right experimental approach, a proportion of these cells can be isolated and grown in laboratories. Researchers can consider modifying their standard laboratory practices and try some different methods for selecting sampling sites, resuscitation approaches, enrichment techniques, or explore the state-of-the-art cultivation methods, for instance in situ and ex situ cultivation. Different DNA sequencing strategies, i.e., WGS, SAG, and MAG, allow researchers to glimpse the genomic contents of underexplored prokaryotes. Overall, it appears that the MAG approach is a more reliable strategy for discovering unculturable microbes. If assembly is performed carefully, MAG methods can be used to generate accurate genome information from uncultivated bacteria as well as underexplored culturable bacteria and will eventually become a standard tool. However, functional-based metagenomics remains expensive and is not affordable on a larger scale. The quality of MAGs is highly dependent on the type and preparation of samples, and MAG requires computational capacity for processing large quantities of sequencing read data and downstream analyses. It is believed that exploring rare prokaryotes is interesting, yet requires due diligence and wise decision-making to overcome all possible challenges.

## Figures and Tables

**Table 1:** Number of 16S rRNA sequences related to prokaryotic phyla. Data were obtained from the NCBI Taxonomy and Silva databases [8].

Phyla and Candidate Phyla	No. of 16S rRNA Sequences		Remarks
	NCBI Taxonomy Database	Silva Database	
**Domain bacteria**			
**Recognized bacterial phylum**			
Acidobacteria	162,567	14,534	Major phylum
Actinobacteria	4,719,261	60,510	Major phylum
Aquificae	23,441	346	
Armatimonadetes	16,593	752	
Bacteroidetes	1,116,583	55,663	Major phylum
Balneolaeota	1589	n/a	
Caldiserica	3630	95	Underexplored culturable, 1 genus, 1 type strain
Calditrichaeota	11,735	275	Underexplored culturable, 1 genus, 1 type strain
Chlamydiae	147,839	450	
Chlorobi	6655	n/a	
Chloroflexi	168,644	9245	Major phylum
Chrysiogenetes	1253	12	Underexplored culturable, 3 genera, 4 type strains
Cyanobacteria	481,641	13,906	Major phylum
Deferribacteres	1968	134	
Deinococcus–Thermus	57,234	948	
Dictyoglomi	296	11	Underexplored culturable, 1 genus, 2 type strains
Elusimicrobia	15,992	435	Underexplored culturable, 2 genus, 2 type strains
Fibrobacteres	8597	751	
Firmicutes	10,435,846	149,757	Major phylum
Fusobacteria	57,398	2216	
Gemmatimonadetes	34,287	21,185	Underexplored culturable, 1 genus, 2 type strains
Kiritimatiellaeota	1149	975	Underexplored culturable, 1 genus, 1 type strain
Lentisphaerae	8098	469	Underexplored culturable, 3 genera, 5 type strain
Nitrospirae	48,424	1297	
Planctomycetes	110,685	9014	Major phylum
Proteobacteria	28,570,321	238,949	
Rhodothermaeota	731	n/a	
Spirochaetes	373,114	4253	Major phylum
Synergistetes	22,879	1152	
Tenericutes	80,247	2561	
Thermodesulfobacteria	4586	n/a	
Thermotogae	22,371	303	
Verrucomicrobia	131,082	4419	
**Domain bacteria**			
**Superphylum unculturable bacteria**			
Abditibacterium	120	155	*Abditibacterium utsteinense* is the first representative of candidate phylum FBP [28]
Abyssubacteria	291	n/a	Bacteria candidate
Acetothermia	3411	165	Bacteria candidate phylum
Aegiribacteria	36	52	Bacteria candidate
Aerophobetes	4393	66	Bacteria candidate phylum
Atribacteria	9087	578	Bacteria candidate phylum
Aureabacteria	106	n/a	Bacteria candidate
Calescamantes	1004	12	Bacteria candidate
Cloacimonetes	10,048	301	Bacteria candidate
Coprothermobacteraeota	811	76	Bacteria candidate
Dadabacteria	3979	169	Bacteria candidate phylum
Dependentiae	1756	580	Candidate bacteria phylum
Desantisbacteria	2580	2	Bacteria candidate phylum
Edwardsbacteria	133	4	Bacteria candidate phylum
Entotheonellaeota	n/a	168	Bacteria candidate
Epsilonbacteraeota	n/a	5422	The class was proposed as phylum [29]
Fermentibacteria	1426	n/a	Bacteria candidate
Fervidibacteria	698	4	Bacteria candidate phylum
Firestonebacteria	770	3	Bacteria candidate
Halanaerobiaeota	n/a	270	Bacteria candidate
Hydrogenedentes	1984	271	Bacteria candidate
Hydrothermae	1987	38	Bacteria candidate phylum
Kryptonia	4416	n/a	Bacteria candidate
Latescribacteria	6535	497	Bacteria candidate
Lindowbacteria	194	1	Bacteria candidate phylum
Margulisbacteria	1455	86	Bacteria candidate
Marinimicrobia	13,480	554	Bacteria candidate
Modulibacteria	n/a	255	Bacteria candidate phylum
Nitrospinae	24,196	2167	Bacteria candidate phylum
Omnitrophicaeota	15,814	507	Bacteria candidate
Patescribacteria	n/a	4521	Bacteria candidate phylum
Poribacteria	3664	49	Bacteria candidate phylum
Rokubacteria	33,383	380	Bacteria candidate phylum
Schekmanbacteria	1628	40	Bacteria candidate phylum
Tectomicrobia	17,004	n/a	Bacteria candidate phylum
Thermosulfidibacteraeota	n/a	3	Bacteria candidate phylum
Zixibacteria	7432	203	Bacteria candidate
Unassigned	6,575,288	54	
Superphylum Candidate Phyla Radiation (CPR)	952	n/a	
**Domain archaea**			
**Recognized archaeal phylum**			
Crenarchaeota	67,854	4611	
Euryarchaeota	307,348	12,957	Major phylum
Korarchaeota	5406	55	Underexplored unculturable
Nanoarchaeota	1689	869	Underexplored unculturable
Thaumarchaeota	39,720	4809	Underexplored culturable, 1 genus, 1 type strain
**Domain archaea**			
**Candidate archaea**			
Aenigmarchaeota	3324	42	Underexplored unculturable DPANN
Altiarchaeota	n/a	935	Underexplored unculturable DPANN
Diapherotrites	1034	1169	Underexplored unculturable DPANN
Huberarchaeota	n/a	909	Underexplored unculturable DPANN
Micrarchaeota	2844	n/a	Underexplored unculturable DPANN
Nanohaloarchaeota	3300	n/a	Underexplored unculturable DPANN
Pacearchaeota	2916	n/a	Underexplored unculturable DPANN
Parvarchaeota	371	201	Underexplored unculturable DPANN
Woesearchaeota	6468	n/a	Underexplored unculturable DPANN
Other unculturable archaea	33,442	2886	Including TACK and Asgard group

n/a: not available.

**Table 2:** Major bacterial genera with more than 100 species.

No.	Phyla	Genus	Total Species ^a^	Total Number of Related Articles ^b^
1	Actinobacteria	*Streptomyces*	848	35,008
2	Firmicutes	*Bacillus*	377	168,001
3	Proteobacteria	*Pseudomonas*	254	162,460
4	Firmicutes	*Paenibacillus*	240	1861
5	Firmicutes	*Lactobacillus*	237	49,320
6	Firmicutes	*Clostridium*	229	54,265
7	Bacteroidetes	*Flavobacterium*	208	5555
8	Actinobacteria	*Mycobacterium*	198	114,210
9	Proteobacteria	*Vibrio*	147	35,798
10	Actinobacteria	*Corynebacterium*	132	19,605
11	Firmicutes	*Streptococcus*	129	142,792
12	Tenericutes	*Mycoplasma*	127	28,075
13	Proteobacteria	*Sphingomonas*	127	3051
14	Proteobacteria	*Burkholderia*	122	11,383
15	Actinobacteria	*Nocardia*	119	7969
16	Proteobacteria	*Rhizobium*	112	24,085
17	Bacteroidetes	*Chryseobacterium*	112	1278
18	Actinobacteria	*Microbacterium*	110	1576
19	Actinobacteria	*Nocardioides*	103	435
20	Proteobacteria	*Halomonas*	102	1411

^a^ Based on LPSN (http://www.bacterio.net/index.html). Subspecies are not counted. ^b^ Scopus data using the respective genus name as the keyword.

**Table 3:** Top ten strains according to patent counts ^a^.

No.	Species	Phyla	Patents	Paper Citations
1	*Corynebacterium glutamicum* ATCC 13032	Actinobacteria	315	478
2	*Staphylococcus aureus* subsp. *aureus* Rosenbach ATCC 6538	Firmicutes	184	610
3	*Synechocystis* sp. PCC 6803	Cyanobacteria	170	4615
4	*Corynebacterium glutamicum* ATCC 13869	Actinobacteria	131	59
5	*Bacillus subtilis* subsp. *spizizenii* ATCC 6633	Firmicutes	125	1292
6	*Escherichia coli* ATCC 25922	Proteobacteria	113	3594
7	*Staphylococcus aureus* subsp. *aureus* ATCC 29213	Firmicutes	108	1809
8	*Brevibacterium flavum* ATCC 14067	Actinobacteria	103	59
9	^b^*Candida albicans* ATCC 10231	Ascomycota	93	488
10	*Escherichia coli* ATCC 8739	Proteobacteria	79	315

^a^ Based on the WFCC Global Catalogue of Microorganisms (GCM) (http://www.wdcm.org/).^b^ *Candida albicans* is yeast.

**Table 4:** Selected monotypic bacteria with genome sequencing information.

Phylum/Family	Monotypic Name	Source	Growth Condition	Genome Size (Mb)	NCBI Genome Accession no.	Ref.
Abditibacteriota/Abitibacteriaceae	*Abditibacter iumutsteinense*	Antartic soil	psychrophile	3.61	GCA_002973605	[28]
Firmicutes/Staphylococcaceae	*Abyssicoccus albus*	deep sea sediment	mesophilic	1.8	GCA_003815035	[65]
Actinobacteria/Micrococcaceae	*Acaricomes phytoseiuli*	predatory mite	mesophile, slow grower	2.4	NZ_AQXM00000000	[66]
Firmicutes/Ruminococcaceae	*Acetanaerobacterium elongatum*	wastewater	mesophile, anaerobe	2.9	NZ_FNID00000000	[67]
Firmicutes/Clostridia	*Acetatifactor muris*	cecum of mouse	mesophile, anaerobe	6.0	NZ_OFSM00000000	[68]
Proteobacteria/Acetobacteraceae	*Acidicaldus organivorans*	hot spring	thermophile	2.89	GCA_000759655	[69]
Actinobacteria/Acidimicrobiaceae	*Aciditerrimonas ferrireducens*	solfataric soil	thermophile	1.18	GCA_001311945	[70]
Chloroflexi/Anaerolineaceae	*Bellilinea caldifistulae*	thermophilic digester sludge	thermophile, anaerobe	3.66	NZ_LGHJ00000000	[71]
Firmicutes/Sporolactobacillaceae	*Caenibacillus caldisaponilyticus*	Acidic compost	thermophile	3.35	GCA_002003465	[72]
Firmicutes/Thermodesulfobiaceae	*Caldanaerovirga acetigignens*	hot spring	thermophile, anaerobe	2.26	GCA_900142995	[73]
Fibrobacteres/Chitinispirillaceae	*Chitinispirillum alkaliphilum*	hypersaline soda lake	mesophile, anaerobic	4.4	GCA_001045525	[74]
Proteobacteria/Hyphomicrobiaceae	*Dichotomicrobium thermohalophilum*	solar lake	thermophile	2.99	NZ_QXDF00000000	[75]
Actinobacteria/Pseudonocardiaceae	*Goodfellowiella coeruleoviolacea*	soil	mesophile	9.3	GCA_000715825	[76]
Proteobacteria/Rhodobacteraceae	*Hwanghaeicola aestuarii*	tidal sediment	mesophile	4.54	GCA_003253995	[77]
Actinobacteria/Pseudonocardiaceae	*Herbihabitans rhizosphaerae*	soil	mesophile	6.64	GCA_004216555	[78]
Proteobacteria/Rhodobacteraceae	*Jhaorihella thermophila*	coastal hot spring	moderate thermophile	3.77	GCA_900108275	[79]
Synergistetes/Synergistaceae	*Jonquetella anthropi*	human cyst	mesophile, anaerobe	1.68	NZ_AGRU00000000	[80]
Actinobacteria/Micromonosporaceae	*Krasilnikovia cinnamomea*	soil	mesophile	7.62	GCA_004217545	[81]
Bacteroidetes/Cytophagaceae	*Leadbetterella byssophila*	cotton waste compost	mesophile	4.06	CP002305	[82]
Proteobacteria/Rhodobacteraceae	*Mangrovicoccus ximenensis*	mangrove forest	halotolerant, mesophile	5.97	GCA_003056725	[83]
Proteobacteria/Beijerinckiaceae	*Methyloferula stellata*	acidic peat soil	psychrophile	4.24	NZ_ARWA00000000	[84]
Proteobacteria/Rhodobacteraceae	*Monaibacterium marinum*	sea water	mesophile	3.73	GCA_900231835	[85]
Bacteroidetes/Flammeovirgaceae	*Nafulsella turpanensis*	soil	mesophile	4.81	GCA_000346615	[86]
Bacteroidetes/Sphingobacteriaceae	*Nubsella zeaxanthinifaciens*	fresh water	mesophile	4.25	GCA_003313335	[87]
Chloroflexi/Anaerolineaceae	*Ornatilinea apprima*	deep well	thermophile, anaerobe,	4.35	GCA_001306115	[88]
Firmicutes/Clostridiaceae	*Oxobacter pfennigii*	rumen of cattle	mesophile, anaerobe	4.51	GCA_001317355	[89]
Bacteroidetes/Sphingobacteriaceae	*Pelobium manganitolerans*	sludge of a mine	mesophile	3.93	GCA_003609575	[90]
Planctomycetes/Planctomycetaceae	*Schlesneria paludicola*	sphagnum peat	mesophile	8.67	GCA_000255655	[91]

**Table 5:** Selected publications and major findings related to metagenomic assembled genomes (MAGs).

Source	Major Bioinformatics Tools	Purpose/Major Findings	NCBI Bioproject Accession no., Unless Stated	Year/Reference
Aquifer	SPAdes, CONCOCT, CheckM	Analyze the genome of Candidate bacterial phylum BRC1.	SRR710274, CP030759	2019 [95]
Hot spring	SPAdes, Kaiju, CheckM	Expanding the understanding (diversity, phylogenetic, and functional) of microbiome in two well-studied hot springs in Kamchatka, Russia.	PRJNA419931	2019 [98]
Deep-sea	BBmap, MetaBAT, CheckM	Recovered 82 MAGs affiliated with 21 different archaeal and bacterial phyla from petroleum seepage. Authors proposed that acetate and hydrogen are the central intermediates underpinning community interactions and biogeochemical cycling.	PRJNA415828, PRJNA485648	2019 [99]
Aquifer	bbduk in the bbmap package, SPAdes, VizBin, CheckM	Assembled the genome of *Rhodoferax* sp. However, the authors were not able to confirm that this bacterium can degrade sulfolane in the contaminated aquifer.	181102 (JGI IMG/ER)	2019 [100]
Soda lakes	BBnorm, MetaSpades, MetaBat, CheckM	Used metagenomics and metaproteomics to provide a comprehensive molecular characterization of a phototrophic microbial mat microbiome.	PRJNA377096	2019 [101]
Lab-scale reactor	CLC de novo assembler, CheckM	Explaining the shifts in microbial community structures using 16S rRNA metagenome, MAGs, and metaproteomic data.	PRJNA471375	2019 [102]
Artificial acid mine drainage	SPAdes, CheckM, ESOM	Describe taxonomy and ecological role of a new order Ca. Acidulodesulfobacterales (Sva0485 clade).	PRJNA517999	2019 [103]
Freshwater	SolexaQA++, Scythe, IDBA-UD, MetaBAT, MASH, MiGA, CheckM	Explain poorly understood Ca. Pelagibacterales (SAR11 clade IIIb).	PRJNA495371, PRJNA214105, PRJNA497294	2019 [104]
Aquifer	IDBA-UD, ggKbase, ABAWACA, ESOM	Reconstruct the genome of Candidate Parcunitrobacter nitroensis (OD1), and Candidate phylum Aminicenantes (OP8).	LBUF00000000, QUAH00000000	2019, 2017 [105,106]
Ocean	Minimus2, BinSanity, CheckM	Reconstruct the genome of 2,631 genomes, as part of Tara Oceans project.	PRJNA391943	2018 [107]
Bay	MEGAHIT, CheckM, RAST, Phylosift, JspeciesWS	Assembled 87 MAGs including archaeal Asgard group (Thorarchaetoa and Lokiarchaeota). Reveal potential microbial interactions.	4761314.3–4761727.3, 4762868.3–4762965.3 (MGRAST)	2018 [108]
Hot spring	IDBA, MaxBin, CheckM	Reconstruct the genome of cyanobacteria *Fischerella thermalis*.	NA382437	2018 [109]
Hot spring	metaSPAdes, CONCOCT, SNAP, CheckM	To relate MAGs’ extracellular electron transfer systems with iron redox-based metabolisms	3300010938 and 3300014149 (IMG/M ER)	2018 [110]
Cow rumen	MetaBAT, dRep, CheckM	Improve the understanding of taxonomic structure of rumen microbiome. To mine novel carbohydrate degrading enzymes.	PRJEB21624	2018 [111]
Offshore station	Refers to original articles	Compare SAG and MAG for samples collected from the same site.	PRJEB21451, LIAK00000000–LIDO00000000	2018 [18,112]
Aquifer	MetaBAT, AMPHORA2	Propose the carbon and nitrogen cycling functions for 71 putative CPR genomes.	SRX2896383	2017 [93]
Metadata obtained from SRA database	CLC de novo assembler, MetaBAT, CheckM, RefineM	Expand the understanding of phylogenetic and genomes of uncultivated bacteria and archaea.	PRJNA34875	2017 [17]
Oil reservoir	EMIRGE, IDBA-UD, ggKbase, ESOM	Describe the metabolic process of candidate phyla.	SRP057267, PRJNA278302	2016 [38]
Aquifer	IDBA-UD, ggKbase, ABAWACA, ESOM	Reconstruct the metabolism to understand the geochemical cycling Construct the CPR genomes and notice that the genomes are small and lacked many important biosynthesis pathways.	PRJNA273161, PRJNA288027	2016, 2015 [33,54]
